# Overcoming the Past-endorsement Criterion: Toward a Transparency-Based Mark of the Mental

**DOI:** 10.3389/fpsyg.2020.01278

**Published:** 2020-06-26

**Authors:** Giulia Piredda, Michele Di Francesco

**Affiliations:** Nets Center, Department of Humanities and Life Sciences, Scuola Universitaria Superiore IUSS Pavia, Pavia, Italy

**Keywords:** cognitive bloat, mark of the mental, consciousness, extended mind, transparency, past-endorsement criterion

## Abstract

Starting from the discussion on the original set of criteria advanced by Clark and Chalmers (1998) meant to avoid the overextension of the mind, or the so-called cognitive bloat, we will sketch our solution to the problem of criteria evaluation, by connecting it to the search for a mark of the mental. Our proposal is to argue for a “weak conscientialist” mark of the mental based on transparent access, which vindicates the role of consciousness in defining what is mental without, however, identifying the mental with the conscious. This renovated link between mind and consciousness, spelled out through the concept of transparency, further develops some of our previous work on the topic (Di Francesco, 2007; Di Francesco and Piredda, 2012) and is partially inspired by Horgan and Kriegel (2008).

## Introduction

Mind-extenders are quite common in contemporary philosophy of mind, and consequently many arguments have been advanced to free our minds from the boundaries of skull and body. Yet, even the most confident mind-extender has to admit that it is necessary to avoid an overextension of the mental (the so-called cognitive bloat, Rowlands, 2009). In this paper, we address this problem in connection with the search for specific Criteria to Avoid the Overextension of the extended mind (let us call them CAOs) proposed by Clark and Chalmers (1998). More specifically, our starting point will be the fourth criterion, the so-called past-endorsement criterion: we think that, by introducing a direct reference to consciousness among the CAOs, this criterion raises important problems, whose solution involves an analysis of the connections between the subpersonal extended vehicles of cognition and the conscious mind of the (extended) subject, which in turn requires an answer to the “mark of the mental” problem.

In the first part of the paper, we will review the problem raised by the past-endorsement criterion since its first appearance in Clark and Chalmers (1998) and retrace its fortunes and misfortunes in the subsequent literature. We will conclude that, even if the solution to the overextension problem offered by the criterion is not satisfying, this portion of the debate is important, in that it suggests the opportunity to further investigate the role of consciousness in distinguishing mental from non-mental resources.

In the second part of the paper, we connect this debate to the search for a mark of the mental, sketching our own solution—which we define as “weak conscientialism,” based on some our previous works (Di Francesco, 2007; Di Francesco and Piredda, 2012; Di Francesco et al., 2016; Di Francesco and Tomasetta, 2017) and partially inspired by Horgan and Kriegel (2008). We draw some conclusions in the last paragraph.

## The Past-Endorsement Criterion as a Solution to the Overextension of the Mind

Following a by now well-established interpretation of the literature on the topic, it is possible to individuate at least three different “waves” in the development of the extended mind theory (Menary, 2010; Gallagher, 2018): the first—in the original version by Clark and Chalmers (1998)—is based on the parity principle^1^; the second—championed by Menary (2007, 2010) and Sutton (2010)—is built around the concepts of integration and complementarity; and the third—still in lively development—starts with enactivism and is connected to the model of the mind inspired by predictive processing framework (Hohwy, 2013; Clark, 2016; Kirchoff and Kiverstein, 2018).

Although time flows, and theories undergo adjustments, it is possible that some “recalcitrant” problems resist the flow of the different waves. In this paper, we start with one of these recalcitrant problems, one that appears already in the seminal paper by Clark and Chalmers and that in the following years—despite the vigorous development of the debate—never attracted much attention, with a few exceptions (e.g., Rupert, 2004; Gertler, 2007; Roberts, 2012).

In the final part of their article, Clark and Chalmers discuss the scope of the extended mind thesis just stated and its potential consequences. To individuate potentially crucial points, they spell out the features involved in the case of *extended belief* they presented the well-known case of Otto’s extended belief stored in his precious notebook. In subsequent literature, these criteria have been dubbed the “glue and trust” criteria (cf. Clark, 2010b); despite their fame, though, there remain unanswered questions regarding both their validity and their role. Here is the first appearance of the criteria:

First, the notebook is a constant in Otto’s life—in cases where the information in the notebook would be relevant, he will rarely take action without consulting it. Second, the information in the notebook is directly available without difficulty. Third, upon retrieving information from the notebook he automatically endorses it. Fourth, the information in the notebook has been *consciously endorsed* at some point in the past, and indeed is there as a consequence of this endorsement. (1998, p. 17, our italics)

The first three criteria—constancy in use, direct availability, and automatic endorsement—appeal to structural or functional features and have been considered as fairly reasonable. Basically, they mimic the normal relation between the conscious mind and its internal subpersonal underpinnings (Di Francesco, 2007). When subpersonal processes give input to the conscious mind, they do it in a systematic and direct way and their content is mandatory. It is assumed by default as a datum, poised for verbal report, reasoning, and so on. In this sense, the first three criteria try to mirror, at the causal level, some important phenomenological properties of the personal mind.

Many critics of the extended mind have highlighted that the first three criteria seem too easily satisfied, such that they would be insufficient to block what has been considered an undesired and implausible proliferation of alleged extended beliefs (Rupert, 2004, p. 401 ff.). Without the “conscious endorsement” requirement, in fact, we should consider as extended beliefs any information coming from a constantly consulted and trusted source: say, for example, a service that provides phone numbers in an efficient and trusted way or some easily accessible web pages. But would it be cognitively plausible to claim that Otto, even before consulting the service, already has beliefs about the phone numbers or about the easily accessible web pages? It seems that posing a more restrictive criterion, that Otto has endorsed a particular content in the past and has thus entertained an occurrent belief about that content, is a good way to avoid the “cognitive bloat” (Rowlands, 2010, p. 93). In other words, if one endorses the extended view of the mind, and accepts that the information stored in Otto’s notebook counts as beliefs, there is a risk of “overextending” the mind: why stop there? Why not also allow all the resources Otto frequently uses among his extended mental states? The idea is that there should be a way to restrict the application of the extension only to *plausible* cases of extended belief.

The overextension of the mind is surely blocked by the fourth criterion Clark and Chalmers put in place: the past-endorsement criterion. According to it, in order for a specific content to be considered one of Otto’s mental states, Otto should have consciously endorsed this content in the past, and this content, say an address, is now stored in Otto’s notebook because of this process of conscious past-endorsement.

Now, while this further criterion eliminates any risk of overextending the mind, one may ask at what cost it does so. As a matter of fact, several problems concerning the past-endorsement criterion have been pointed out. Just after having presented it, Clark and Chalmers themselves (1998, p. 17) recognized the problematic status of this criterion when they observed that non-extended beliefs may be acquired via non-conscious processes, and imposing the additional conscious endorsement criterion only to extended beliefs would be at least arbitrary. On a more general note, the role assigned to consciousness by this criterion does not seem in line with the spirit of the extended mind framework, which tries to undermine the privilege to internal processes, like consciousness. This point has been made explicitly by Rupert (2004):

If an extended (or any) belief requires conscious endorsement in order to be a genuinely held belief, and conscious endorsement is ultimately an internal process […], then the traditional subject is privileged in a deep sense, after all (p. 404).

There are several observations deriving from this situation, and some of them will lead us toward the next section, dedicated to the search for a mark of the mental as a solution to the problem of the overextension of the mind.

The first concerns the role of consciousness: even if it is true that we acquire and form beliefs also unconsciously, and it is implausible to assume two different generation processes for extended and non-extended states, it is possible to interpret the emergence of the topic of consciousness in the debate about the extended mind as non-arbitrary^2^. The idea is that the fourth criterion is perhaps too strong, and unacceptable, as it is, but we feel that the discussion it raises is important in that it suggests a role for consciousness in defining what is properly mental. We will return to this notion.

Second, this is not the only occasion in which Clark seems to have a prudential attitude and defend the priority of the “organism-centered,” even if not “organism-bound,” cognition (Clark, 2008, p. 123). In the further literature on extended mind, Clark and Chalmers have been criticized for this attitude, defined as “too Cartesian,” which would be entailed—according to many—by the parity principle (cf. Sutton, 2010, in Gallagher, 2018, p. 430; Wheeler, 2010).

Third, another dimension that is missing in the first three criteria, while well represented by the fourth, is a historic dimension: that is, the fact that the agent has been acquainted with some contents and—partly because of this—we could attribute these contents to him. However, a historic solution is not the only available. Also, we will offer an alternative functional solution.

The curious thing is that, although the debate on the extended mind has flourished in the last few decades, a thorough discussion on the issue of criteria evaluation—and particularly the status of the fourth criterion—is still lacking^3^. As we have seen, the problematic status of this criterion was promptly acknowledged by Clark and Chalmers (p. 17), who, after warning the reader, left the criterion in a sort of “theoretical limbo.” As noted before, a full-blown criticism of the criterion was later developed by Rupert (2004). In the further literature, the criterion was at times mentioned, at times it was missing (Menary, 2010, p. 424; see Clark, 2010b, p. 50; Gallagher, 2018), while in Clark (2008) the fourth criterion was treated as problematic, but nevertheless relevant: “the ‘past conscious endorsement’ criterion looks too strong. On the other hand, to drop this requirement opens the floodgates to […] an unwelcome explosion of potential dispositional beliefs” (p. 96). However, as far as we know, the topic has never been fully elaborated by Clark and Chalmers in their subsequent works.

In this paper, we will get our chance to sketch a solution to this discussion, connecting the missing (or underestimated) debate on the fourth criterion to the fundamental issue of the mark of the mental. Our “sketch” will focus on the role of transparent access (Clark, 2004, 2008; Wheeler, 2019)—a fundamental feature of consciousness—in defining what is mental, thus contributing to the issue of the mark of the mental. In our view, the lack of analysis dedicated to the past-endorsement criterion is revealing of a missing analysis of the relation between the extended mind and the role of consciousness. We believe that, within the extended mind framework, the lack of a serious analysis of the role of consciousness in marking the mental opens the door to the risk of overextension and thus leaves the entire framework wanting. A proper treatment of these important points of connection is due.

## The Mark of the Mental

### From the Criteria to Avoid Overextension (CAOs) to the Mark of the Mental

The connection between the CAOs and the mark of the mental is, in a sense, direct. The four CAOs were introduced by Clark and Chalmers to avoid mental overextension, and having a mark of the mental seems an immediate way to succeed in this goal. Imagine that we want to know if a certain state, event, or process is a mental item. If we had a mark of the mental, we would only have to check whether the item in question meets the criteria set by the mark.

Among the criticisms addressed to the first wave of extended mind, the lack of such a mark of the cognitive or mark of the mental has been one of the most significant (see Adams and Aizawa, 2001, 2008; Piredda, 2017 for discussion)^4^. The idea is that “causal coupling” alone, even if constrained by the three “glue and trust” criteria, is not sufficient to individuate genuine examples of *cognitive* or *mental* activity: one would need a mark of the mental in order to discriminate the cases to be rightly counted as such.

While Adams and Aizawa (2001, 2008) provide a mark of the cognitive based on the notion of intrinsic content, Clark and Chalmers (1998) do not seem to provide such a mark. It is true that the problem of the mark was not mentioned in the 1998 paper, but Clark later acknowledged this problematic point and has dedicated some thoughts to it (Clark, 2008, 2010a,b,c). His ideas about the topic are oriented to a purely minimalist and functionalist position—an interesting approach, but one that is not able to solve on its own all the problems of overextension.

First of all, in Clark’s view, what is cognitive or non-cognitive is not the single component of a certain process, but rather the process as a whole, which must be involved in supporting intelligent behavior:

What makes a process cognitive […] is that it supports intelligent behavior (Clark, 2010a, p. 92).

The study of mind might […] need to embrace a variety of different explanatory paradigms whose point of convergence lies in the production of intelligent behavior (Clark, 2008, p. 94)^5^.

Thus, according to Clark, the processes could in principle be implemented by various kinds of substances (biological or artificial substrates, as well as external resources), because what defines something as cognitive or mental is neither the substance that realizes it nor the detailed causal dynamics that characterize its workings. In this sense, cognitive or mental processes in the extended framework are individuated on the basis of coarse or common-sense functional considerations concerning cognitive processes such as memory, understanding, categorization, reasoning, etc.

It is the coarse or common-sense functional role that, on this model […], displays what is essential to the mental state in question (Clark, 2008, p. 89).

The reference to the causal relationship as the starting point of the analysis on mental reality is, after all, at the base of the (extended) functionalist intuition:

What makes some information count as a belief is the role it plays, and there is no reason why the relevant role can be played only from inside the body (Clark and Chalmers, 1998, p. 14).

The only available mark of the mental in the extended mind approach concerns the functional analysis of the resource in a given context. The idea is that in the extended mind approach the mark of the mental is not something already given; rather, it is something one discovers, starting from an intuitive and shared idea of what a mental process is. The minimal and operational mark derived from this “commonsense functionalism,” however, seems to be just a pragmatic instrument that does not characterize the mental in a substantive manner and is limited to granting a “cognitive” and/or “mental” status to those parts of a system that play a central role in a “recognizably cognitive process.”

These are cases when we confront *a recognizably cognitive process*, running in some agent, that creates outputs (speech, gesture, expressive movements, written words) that, recycled as inputs, drive the cognitive process along. In such cases, any intuitive ban on counting inputs as parts of mechanisms seems wrong (Clark, 2008, p. 131, our italics).

It is, above all else, a matter of empirical discovery, not armchair speculation, whether there can be a fully fledged science of the extended mind (Clark, 2008, p. 95).

The problem is that such a minimal and operational mark of the mental is very unlikely to save the model from the risk of overextension. We believe that it is necessary to deepen the analysis of what is mental, referring to the role of consciousness and of personal level in depicting an adequate mark. The same attitude is shared by authors that have dealt with the mark of the mental or the problem of criteria (such as Rupert, 2004; Gertler, 2007; Rowlands, 2009; Roberts, 2012; Adams and Garrison, 2013; Varga, 2018), although we do not have the opportunity to discuss them here.

In the next paragraph, we will sketch our own solution, starting from the rediscovery of the role of consciousness in marking the mental and based on the notion of transparency (see Clark, 2004, 2008; Wheeler, 2019). It is developed from some of our previous works on the topic (Di Francesco and Piredda, 2012; Di Francesco et al., 2016; Di Francesco and Tomasetta, 2017) and from a valuable discussion of the mark of the mental by Horgan and Kriegel (2008), recently revisited by Gallagher (2017). Lastly, we will consider some general conclusions concerning the extended mind framework that derive from it.

### The Mark of the Mental: Some Preliminary Thoughts

To sum up, we find ourselves in a situation in which the search for Criteria to Avoid Overextension (CAOs) ends in a dilemma. On the one hand, it seems that keeping the first three criteria and rejecting the fourth—the past-endorsement criterion—will open the extended mind framework to a potentially undesired proliferation of extended beliefs. On the other hand, keeping all four criteria has proven problematic for the extended mind model, as it would imply an overly privileged position for consciousness in deciding what counts as a belief—a position not applicable to internal states.

A straightforward alternative to the problem of finding the right criteria, as already mentioned, is to offer a proper mark of the mental. This is, however, no simple task, and many proposals have already been made on the topic. The particular perspective we wish to take on this subject comes from an acknowledgment of the importance of the role that the past-endorsement criterion has had to play in this story. We think that the fluctuating presence of the past-endorsement criterion in the literature on the extended mind indicates something interesting about the role it was meant to play. Our contribution to the debate would be to sketch a possible version of the mark of the mental that also has the merit of defining the suspended status of the conscious past-endorsement criterion, thereby establishing an often neglected issue. Before introducing our proposal, some preliminary—though simplified—considerations are in order.

The battlefield of the mark of the mental has been traditionally divided into two areas: on the one hand, broadly following Franz Brentano, it has been claimed that *intentionality* is what mainly characterizes the mental domain. On the other hand, *consciousness* has been considered the distinctive feature of our mind. Now, on which side of the field should a mind-extender line up?

If one goes for the intentionalist side, one has to remember that the distinction between intrinsic and derived intentionality is not necessarily available to the mind-extender (see Searle, 1992; Clark, 2005; Dennett, 2009). Moreover, if one lacks the means to distinguish between the two, it would be difficult to distinguish between natural and artificial intentional systems, as long as they entertain intentional states.

On the other hand, the conscientialist option should be further specified. One could think that phenomenal consciousness is what distinctively characterizes our mental experience, but this is not the only possible interpretation of the role of consciousness in defining a mark of the mental. Consciousness is also a particular way through which we have access to our mental states, one that, at least since Descartes, has played a fundamental role in the construction of theories of mind. We seem to have direct access to our mental states, and we act according to them without questioning whether they are really ours. This condition is something very similar to what the “glue and trust” criteria—along with the conscious past-endorsement criterion—attempt to grasp. Even if it is implausible to claim that every single mental state—say, a belief—has been consciously endorsed before entering our mind, we believe the reference to the role of consciousness, and the particular way we have access to some contents of our mind, to be nevertheless meaningful. Even if the conscious past-endorsement criterion has to be rejected, its pointing to consciousness may represent an appropriate suggestion to follow.

This is the intuition we intend to follow in the remainder of this paper: to rediscover the central role of consciousness in accounting for the specific features of our mind. In so doing, we will conclude that the past-endorsement criterion is wrong, but that it nevertheless indicates the right direction to follow in acknowledging a fundamental role to consciousness in defining what can count as mental.

Our path will be divided into two steps: the first concerns the form, or the structure, of the mark of the mental; the second regards its content.

Usually, when we think of the form of the mark of the mental, we imagine a feature or a set of features that, if possessed by a process or a state, unmistakably qualify that process or state as mental. They can be considered as necessary and sufficient conditions for mentality. This way of looking at the problem makes the quest for the mark of the mental even more difficult that it already is, as it demands a great deal of any theory of the mental^6^. However, the individuation of necessary and sufficient conditions is not the only possible kind of a mark of the mental and, of the other possible candidates, we will rely on the “two-layer” mark of the mental by Horgan and Kriegel (2008)^7^, based on the prototype theory (Rosch, 1973)^8^.

According to Horgan and Kriegel (2008), the concept “mental” is organized as a prototypical concept (Rosch, 1973). If this is so, there are some prototypical mental states that constitute the standard cases, and other states that can be defined as mental in virtue of a relationship they entertain with the prototypical cases. In Horgan and Kriegel’s view, the prototypical mental states are phenomenally intentional states^9^, defined as “uncontroversially, unquestionably, paradigmatically, prototypically mental” and “other mental states count as mental only when, and insofar as, they bear the right relationship to phenomenally intentional states” (p. 8). An interesting aspect of this view is that, depending on the intensity of the relation with the prototypical mental states, the mentality of the other states comes in degrees, admitting “gray areas in which there is no deep fact of the matter as to whether a given state is mental or not.” This is the reason why Horgan and Kriegel speak of a “two-layer” mark: the first layer is composed of phenomenally intentional states, “the only ones that qualify as mental in and of themselves and regardless of any relationship they might bear to any other state,” while the second layer is composed by all “the relevant states […] that are causally integrated in the right way within larger systems that feature phenomenally intentional states” (p. 10).

Now, while Horgan and Kriegel choose phenomenal intentional states as the prototypical mental states, it is of course possible to select other states as prototypical and still keep the prototypical structure of the mark of the mental. This is what we propose later in this work. The time is now ripe to present our proposal, dedicating some thoughts to the content of the mark of the mental.

### Sketches for a Transparency-Based Mark of the Mental

In the last section, we specified that in our view the mark of the mental should not be considered as a necessary condition that an agent’s mental states have or do not have, but rather as a prototypical concept to which it is possible to be nearer or further (this idea is inspired by Horgan and Kriegel, 2008). Having established this, we can now tackle the question of the content of the mark of the mental.

Our proposal is that (1) conscious states are the prototypical mental states and (2) some unconscious/subpersonal states can also legitimately be considered as mental: this happens when they have a particular relation with conscious states (Di Francesco and Piredda, 2012; Di Francesco et al., 2016; Di Francesco and Tomasetta, 2017). Condition (2) will also apply for some extended putative mental states.

The question is now: how is such a relation to be specified? We suggest that the main characteristic to describe this relation is “transparent access.” We rely on the conception of transparency developed by Clark (2004, 2008) and Wheeler (2019), inspired by the phenomenological tradition (see Heidegger, 1927; Merleau-Ponty, 1945). This is a conception of “phenomenological transparency” in the sense that it depends on what is perceived and experienced by the agent. In the famous example by Heidegger, the skilled carpenter has no conscious recognition of the hammer in use: “when we skilfully manipulate equipment in a hitch-free manner, we have no conscious apprehension of the items of equipment in use as independent objects, that is, as something like identifiable bearers of determinate states and properties” (Wheeler, 2019, p. 859). Tools in use become thus phenomenologically transparent. Speaking of the body, Clark writes:

At such moments, the body has become “transparent equipment” (Heidegger, 1927/1961): equipment (the classic example is the hammer in the hands of the skilled carpenter) that is not the focus of attention in use. Instead, the user “sees through” the equipment to the task in hand. When you sign your name, the pen is not normally your focus (unless it is out of ink etc.). The pen in use is no more the focus of your attention than is the hand that grips it. Both are *transparent equipment*. (Clark, 2008, p. 10, our italics)

This conception of transparency is thus construed in analogy with the transparency in tool use and in technology. In this context, a process (even an “extended” process) is taken to be transparent if it is invisible to the subject, who uses it in a fully unconscious and automatic way; yet, the results of the process must be accessible to the subject’s consciousness (even if the process itself is not). In this way, we achieve a strengthening of the link between the mental and the conscious (Di Francesco and Piredda, 2012, Chap. 5).

Our idea is to take transparent access to consciousness as fundamental for mentality: being transparently accessible by consciousness, or being sufficiently integrated with a mental state which is transparently accessible by consciousness, rather than being internal to the skull, is what makes something mental. In this sense, transparency expresses the idea of a strong integration between the subject’s conscious mind and her other mental processes—where integration is to be considered a relation of coupling in which a component’s output is recycled as input from the other component—as in the case of the output of an unconscious process that is used as input from a conscious one.

There is no special magic associated with direct physically wired links between components. The differences between links forged by nerves and tendons, by fiber-optic cables, and by radio waves are relevant only insofar as they affect the timing, flow, and density of informational exchange. These latter factors are relevant, in turn, because they affect the nature of our relationship with the various kinds of tools, equipment, and subsystems. If the links are sufficiently rich, fluid, bidirectional, fast, and reliable, then the interface between the conscious user and the tool is liable to become transparent, allowing the tool to function more like a proper part of the user. (Clark, 2003, p. 103)

Transparency brings about a sort of direct access of the subpersonal content to consciousness—in the sense that at the phenomenological level the given content is directly available to the conscious/personal mind of the subject. In other cases, transparency plays a less direct but still relevant role:

Applied to the mark of the mental issue, this allows us to regain, for instance, those subpersonal states that are seemingly endowed with a representational content (e.g., Marr’s 2^1^/_2_-D sketch, or perceptual processing in the ventral pathway) and, though being not directly accessible by the personal mind, are sufficiently integrated with personal processes. In sum, (derived) mentality requires integration between conscious and unconscious. (Di Francesco et al., 2016, p. 46)

According to this view, Marr’s 2^1^/_2_-D sketches represent a good example of how integration—together with the transparency of the final output—may drive the individuation of derived *internal* mentality. Analogously, there may be extended processes that involve states or processes that, though not strictly transparent themselves, can be considered as cases of derived *extended* mentality in virtue of their being strongly integrated with other (extended, mental) processes. Examples of this kind could be the processes Otto uses in order to retrieve his extended beliefs on the notebook and some processing of an external cognitive prosthesis at work (see Vold, 2015, pp. 26–27).^10^

The fact that we believe that the prototypical mental cases are conscious states is not to say that *only* conscious states are mental—which would be a *strong* conscientialist proposal—but just to submit that mental states, conscious or unconscious, should stay in the right sort of relation to the personal/conscious mind. For this reason, we have qualified our proposal as “weak conscientialism.”

Interestingly, a mark of the mental based on the degree of transparency offers the possibility of providing a continuous and somehow measurable mark. So it could be possible, in principle, to elaborate a “scale of mentality” based on a multidimensional matrix. One proposal in this sense has been advanced by Heersmink (2012) concerning mind–artifact relations. The dimensions considered include reliability, durability, trust, procedural and representational transparency, individualization, bandwidth, speed of information flow, distribution of computation, and cognitive and artifactual transformation. In this way, the concept of mentality could be considered a nuanced and more inclusive concept. Of course, depending on how far one is willing to stretch the concept in the direction of less prototypical cases, the concept can—to greater or lesser degrees—be kept in line with our intuitive comprehension of it.

A last important point regards how our criterion works in several examples of putative extended mentality. In particular, we would like to test it in two cases: the already mentioned case of the extremely efficient electronic phone book imagined by Rupert (2009) and previously mentioned this paper and the case of some contents accessible through Google, often used as a possible counterintuitive consequence of the extended mind framework.

According to our reasoning, we believe that, in the case of the very efficient electronic phone book, we could consider its contents as plausible examples of extended beliefs if the first three criteria indicated by Clark and Chalmers are satisfied and if the electronic phone book is perceived by the agent as a transparent resource. This feeling of transparency can be continuous, or it may change over the course of the agent’s life—it is possible that when we buy a new electronic tool, for example, there is a transition period during which we are more familiar with the old tool, but then we gain familiarity with the new one, which “magically” becomes transparent. Thus, if the instrument “disappears” when we use it, it is legitimate to consider it as a piece of our extended mind.

The case of the contents of Internet pages accessed through Google is entirely different in our view. In fact, even if we could imagine the day in which we can access Internet pages by wearing a pair of Google glasses or in other very immediate and direct ways, there still is a criterion that seems not to be satisfied by this kind of resource: that is, the automatic endorsement, according to which the agent (say, Otto), upon retrieving information from the notebook, automatically endorses it. It is very unlikely to imagine that we would endorse any possible content transparently and immediately retrievable from the Internet, and this is a good reason—at least for the moment—to leave Internet pages out of our extended mind.

So, in conclusion, what our criterion of “transparent access” adds to the first three criteria is a phenomenological condition on how we “live” our relation with the resource in question. It is a functional–phenomenological condition, quite far from the historic condition proposed by Clark and Chalmers.

As we have seen, the notion of transparency we have in mind has several components: immediacy, direct availability, and integration, and in our view it should help us discriminate mental from non-mental resources. The reference to transparent access to mark the mental is useful to discriminate the mental from the non-mental from both sides: from the inside, to distinguish subpersonal states that “deserve” the label “mental” (for example Marr’s 2 ^1^/_2_–D sketches) from states that are very far from the mind (e.g., low-level neurophysiological states); from the outside, to distinguish plausible cases of extended mental states (for example, Otto’s extended mental states) and less plausible ones (e.g., transparently retrievable contents of any Google page).

By putting these two steps (form and content of the mark of the mental) together, we are able to sketch a solution to the problem of criteria and the mark of the mental. On the one hand, the notion of mental can be extended to incorporate subpersonal phenomena, provided that these are somewhat integrated with conscious processes (as shown by the concept of transparency). On the other hand, in accordance with this criterion for the mental, we claim that the subpersonal approach has to be integrated with reference to the personal level, in contrast with the approaches that fail to appreciate the link between personal and subpersonal.

## Concluding Remarks

The weak conscientialist mark of the mental we have just sketched, which gives a central role to consciousness and personal mind, seems to concede much to an internalistic picture of the mind. From this point of view, our proposal seems to share with Horgan and Kriegel (2008) and Farkas (2012) the downplaying of the philosophical significance of the extended mind hypothesis. This might be true at the metaphysical level (the paradigm shift imposed by the extended mind hypothesis does not affect the centrality of the personal mind), but on the methodological and anthropological levels, things are different. On the methodological side, only time will tell what progress an externalist investigation of mental states can provide. On the anthropological side, the consideration of human beings as natural-born cyborgs can lead us to review our vision of human beings, with evident philosophical and ethical follow-ups.

In particular, we think that the significance of the extended mind model is not limited to the metaphysical or epistemological evaluation of the mental (that, even by Clark’s admission, could be in principle non-provable on empiric grounds, see Clark, 2011). The extended mind model is worth analyzing also for its anthropological and cultural significance: it helps us recognize our fundamental debts toward the external environment in constructing our habitual everyday lives. We think that acknowledging our nature as “natural-born cyborgs” (Clark, 2003) helps us show that extended cases of mentality should not be considered as such extravagant and uninteresting cases of mentality as Horgan and Kriegel seem to think (2008, p. 22): rather, the important way in which we delegate to external resources so much of our thought and private information testifies to the importance of these material external resources in the construction and maintenance of our thoughts and memories. Moreover, disregarding this phenomenon could represent a serious shortcoming for a contemporary theory of the (extended) mind.

Our solution could perhaps be labeled as weakly Cartesian, because of the central role of the conscious mind. However, at the same time it allows moderate extensions of the mind—and paves the way for the philosophical anthropology of the “natural born cyborgs” proposed by Clark (2003)—a view of human nature that we take as one of the most significant by-products of the adoption of the extended mind stance.

## Author Contributions

GP and MD conceived this manuscript. GP wrote the first draft of the manuscript. Both authors contributed to manuscript final version, its revision, and they read and approved the submitted version.

## Conflict of Interest

The authors declare that the research was conducted in the absence of any commercial or financial relationships that could be construed as a potential conflict of interest.

## Footnotes

1 “If, as we confront some task, a part of the world functions as a process which, were it done in the head, we would have no hesitation in recognizing as part of the cognitive process, then that part of the world is part of the cognitive process” (Clark and Chalmers, 1998, p. 8).

2 Interestingly, Clark has explicitly defended internalism regarding consciousness (see Clark, 2009, 2012). The debate about extended consciousness is still open (e.g., Lycan, 2002; Vold, 2015; Kirchoff and Kiverstein, 2018; Chalmers, 2019; Manzotti, 2019), and the possibility of extending consciousness would bring completely different solutions to the problem solved by the past-endorsement criterion. Unfortunately, discussing these alternative possibilities would lead us astray from the topic of this article.

3 Among the few exceptions is Gertler (2007). In her paper, she finds a way to block the overextension of the mind by blocking the extended mind itself, criticizing one premise of the argument for it. The result is an argument for a “narrow mind,” according to which the mental is restricted to the conscious. While we find her point of view undoubtedly interesting, we do not agree with her conclusion—we would like to find a way to resist the undesired overextension of the mind, maintaining the existence of unconscious mental states and processes.

4 In this paper, we shall use “mark of the mental” and “mark of the cognitive” as essentially synonymous expressions. The reason for this apparently objectionable choice is that there is no firmly established use of these two expressions in the debate on the extended mind. Generally speaking, “mental” has a broader meaning, and “cognitive” may refer to a subset of mental phenomena. Another difference (aligned, perhaps, with Clark and Chalmers’ approach) is that “cognitive” may be reserved for subpersonal “intelligent” processing (as in the Tetris example) and “mental” for (potentially) conscious states (such as Otto’s and Inga’s beliefs). Most of the literature on the extended mind has focused more on the mark of the cognitive than on the mark of the mental. While the problem of clearly distinguishing between the two lies beyond the scope of this paper—and actually concerns most of the literature on the extended cognition/mind debate—we believe that the terminological choice between “cognition” and “mind” will depend, at least in part, on the philosophical taste and tradition of the author: a philosopher of cognitive science is more likely to talk about cognition, while it is more probable that an analytic philosopher, or a follower of the phenomenological tradition, or even a follower of radical enactivism, will talk about the mind and the mark of the mental. The distinction appears to be more sociological than metaphysical, so to speak. Nevertheless, we also believe that, from a substantial point of view, the debate on the “mark of the cognitive” developed in the literature on the extended mind, to which we refer in this section, is extremely relevant for the issue of the mark of the mental: the positions of Clark, Adams, and Aizawa on the mark of the cognitive are easily transferable to the issue of the mark of the mental.

5 By the way, these two quotes represent a very clear example of the relaxed use of “mind” and “cognitive” in the debate about the extended mind. Another example is the following quote from Gallagher: “The strict distinction between causality and constitution is closely tied to the idea that there is a ‘mark of the mental’ (a way to determine what processes count as cognitive and what processes do not)” (2017, p. 7).

6 As far as we know, the proposals by Adams and Aizawa (2008) and by Rowlands (2009) regarding the mark of the cognitive adopt this intuition, and both have encountered considerable problems.

7 More recently, Kriegel (2017) has proposed to interpret “mental” as a natural kind concept, having a necessary and sufficient underlying nature. In any case, already in Horgan and Kriegel (2008), fn. 24, p. 370 it is specified that “there is no real tension between being a natural kind concept and being a prototype concept. A natural kind prototype concept would be one for which the relevant relationship non-prototypical instances would have to bear to prototypical ones is that of (probably exact) similarity with respect to underlying nature.”

8 Another possibility is to adapt Gallagher’s “pattern theory of the self” to the case of the mark of the mental (Gallagher, 2013, 2017). We find this proposal unattractive as, in our view, more than a theory of the mark of the mental, this would qualify as a theory concerning the non-existence of a mark of the mental, and we would like to believe that—also being a natural/biological category—having a mind could be somehow described in a substantive manner.

9 “The phrase “phenomenal intentionality” denotes a kind of intentionality that phenomenally conscious states exhibit and moreover exhibit precisely *in virtue* of being phenomenally conscious states, that is, in virtue of their specific phenomenal character” (Horgan and Kriegel, 2008, pp. 5–6).

10 Another interesting case is the one of “language scaffolding,” when for example we are writing a paper and we rely on a series of external resources in order to get our job done. Some of these resources could be considered transparent and some others not, but they are nevertheless so intimately integrated into the extended cognitive process to be considered also part of the extended mental system (see Clark, 1997, pp. 206–207). We would like to thank a reviewer for having pushed us to make clear the role of integration, together with transparency, not only in the cases of non-conscious *internal* mentality but also in the cases of *extended* mentality.
